# Economic evaluations of next-generation sequencing for targeted therapy in non-small cell lung cancer: a systematic review

**DOI:** 10.3389/fpubh.2026.1896681

**Published:** 2026-09-08

**Authors:** Zexiang Dai, Wentao Ye, Jin Zhou, Lei Tian

**Affiliations:** School of International Pharmaceutical Business, China Pharmaceutical University, Nanjing, China

**Keywords:** cost-effectiveness analysis, next-generation sequencing, non-small cell lung cancer, pharmacoeconomics, systematic review

## Abstract

**Aim:**

This study aimed to systematically review model-based economic evaluations of next-generation sequencing for guiding targeted therapy in non-small cell lung cancer.

**Methods:**

We searched PubMed, Embase, the Cochrane Library, Web of Science, China National Knowledge Infrastructure, VIP Database, and Wanfang Database from inception to March 20, 2026, to identify economic evaluations of next-generation sequencing for molecular testing in non-small cell lung cancer. Two reviewers independently screened studies, extracted data, and assessed reporting quality using the Consolidated Health Economic Evaluation Reporting Standards 2022 checklist. A qualitative synthesis was conducted to summarize study characteristics, model structures, testing strategies, cost-effectiveness results, and sensitivity analyses.

**Results:**

Eighteen model-based economic evaluations were included. Seven modeling approaches were identified, among which hybrid models combining a decision tree with a partitioned survival model were most frequently used, followed by decision tree–Markov models. Most studies evaluated next-generation sequencing against single-gene testing or sequential molecular testing, although the tested genes, panel sizes, comparator strategies, and treatment pathways varied substantially across studies. Epidermal growth factor receptor and anaplastic lymphoma kinase were the most commonly included biomarkers. Overall, most studies suggested that next-generation sequencing was cost-effective compared with conventional testing strategies, but several studies from Asian settings reported unfavorable cost-effectiveness results. Key drivers of model results included treatment costs, time horizon, testing strategy, prevalence of actionable alterations, and turnaround time.

**Conclusion:**

Current evidence suggests that NGS-guided strategies may improve health outcomes in NSCLC, but their economic value remains highly context-dependent. Cost-effectiveness is influenced not only by testing strategies, but also by the availability, accessibility, and cost of matched targeted therapies, as well as turnaround time and other real-world implementation factors. Future evaluations should therefore assess NGS within the broader molecular testing–treatment pathway and better reflect real-world clinical practice.

## 1 Introduction

Lung cancer remains one of the most frequently diagnosed malignancies and a leading cause of cancer-related mortality worldwide (1). Non-small cell lung cancer accounts for approximately 80–85% of all lung cancer cases, and a substantial proportion of patients are diagnosed with locally advanced or metastatic disease, for which the overall prognosis remains poor (2). In recent years, advances in molecular classification and precision oncology have shifted the treatment paradigm of non-small cell lung cancer from histology-based treatment alone toward biomarker-guided individualized therapy (3).

Non-small cell lung cancer is characterized by substantial molecular heterogeneity. The identification of actionable genomic alterations, including alterations in *EGFR, ALK, ROS1, BRAF, MET, RET, NTRK, KRAS*, and *ERBB2*, has become central to clinical decision-making in advanced disease (4, 5). In molecularly selected patients, targeted therapies for several of these alterations have demonstrated superior progression-free survival and objective response rates compared with conventional chemotherapy, often with clinically meaningful improvements in disease control (6). Therefore, accurate, timely, and comprehensive molecular testing is now an essential component of the diagnostic and therapeutic pathway for advanced non-small cell lung cancer (7).

Conventional single-gene testing and sequential testing remain clinically useful for detecting high-prevalence biomarkers such as EGFR mutations and ALK rearrangements (8, 9). However, as the number of actionable biomarkers continues to expand, sequential single-gene testing may lead to tissue exhaustion, prolonged turnaround time, and missed rare genomic alterations. Next-generation sequencing enables the simultaneous detection of multiple types of genomic alterations, including single-nucleotide variants, insertions and deletions, gene fusions, and copy-number alterations, within a single assay. It can therefore improve the efficiency of molecular profiling and facilitate the selection of matched targeted therapies (5, 10, 11). International guidelines and expert recommendations, including those from the National Comprehensive Cancer Network (NCCN), the European Society for Medical Oncology, and the College of American Pathologists/International Association for the Study of Lung Cancer/Association for Molecular Pathology, recommend broad molecular testing in patients with advanced or metastatic non-small cell lung cancer and highlight the role of next-generation sequencing in comprehensive biomarker assessment.

Despite its clinical potential, the economic value of next-generation sequencing is influenced by multiple factors, including testing strategy, panel scope, prevalence of actionable alterations, turnaround time, access to matched therapies, drug prices, and reimbursement policies (12, 13). Therefore, this systematic review aimed to synthesize model-based economic evaluations of next-generation sequencing for detecting genomic alterations and guiding targeted therapy in non-small cell lung cancer, with a focus on study design, model structure, testing strategies, economic outcomes, and sensitivity analyses. The findings may help clarify the current evidence base, identify methodological limitations, and inform future economic evaluations and molecular testing strategies in non-small cell lung cancer.

## 2 Methods

### 2.1 Eligibility criteria

This systematic review included model-based health economic evaluations of next-generation sequencing (NGS) for detecting genomic alterations and guiding targeted therapy in patients with non-small cell lung cancer (NSCLC). Eligible studies included cost-effectiveness analyses, cost-utility analyses, and cost–benefit analyses. The target population was patients with NSCLC who underwent NGS-based molecular testing to guide the use of targeted therapies. The intervention was defined as any NGS-based testing strategy, without restrictions on panel size, sample source, or testing pathway. The main outcomes of interest were quality-adjusted life-years (QALYs) and incremental cost-effectiveness ratios or incremental cost-utility ratios (ICERs/ICURs). Studies were excluded if they were published in languages other than Chinese or English, were duplicate publications, conference abstracts, reviews, commentaries, or editorials, or did not report relevant economic outcomes. Studies that only conducted cost analyses or budget impact analyses, without cost-effectiveness or cost-utility outcomes, were also excluded. In addition, studies with target populations or interventions inconsistent with the scope of this review were excluded.

### 2.2 Search strategy

A comprehensive literature search was conducted in PubMed, Embase, the Cochrane Library, Web of Science, China National Knowledge Infrastructure (CNKI), VIP Database, and Wanfang Database from database inception to March 20, 2026. The search strategy combined controlled vocabulary and free-text terms related to NSCLC, NGS, and economic evaluation. Key search concepts included “non-small cell lung cancer,” “non-small-cell lung carcinoma,” “next-generation sequencing,” “NGS,” “cost-effectiveness,” “cost-utility,” “cost–benefit,” “economic evaluation,” and “pharmacoeconomics.” Corresponding Chinese terms were used for searches of Chinese-language databases. The search strategy was adapted for each database according to its specific indexing terms and search syntax.

### 2.3 Study selection and data extraction

This review was conducted in accordance with the Preferred Reporting Items for Systematic Reviews and Meta-Analyses (PRISMA) guidelines (14). After removing duplicates, two reviewers independently screened the titles and abstracts of all retrieved records. Studies that were clearly irrelevant were excluded. The full texts of potentially eligible studies were then assessed according to the predefined inclusion and exclusion criteria. Any disagreement between the two reviewers was resolved through discussion or consultation with a third reviewer.

Data were extracted independently by two reviewers using a predefined data extraction form. The extracted information included: (1) basic study characteristics, including first author, year of publication, country or region, and study perspective; (2) methodological characteristics, including study type, target population, intervention and comparator strategies, model type, time horizon, and cycle length; and (3) economic evaluation results, including base-case results, sensitivity analyses, scenario analyses, and main conclusions. To improve comparability across countries and study years, reported willingness-to-pay (WTP) thresholds were standardized to 2023 international dollars (Int$) using inflation adjustment and purchasing power parity conversion, based on the original currency and reference year reported in each study. When relevant information was unclear or not reported, no assumptions were made, and the information was recorded as not reported (NR). The extracted data were cross-checked by the two reviewers, and discrepancies were resolved through discussion or adjudication by a third reviewer.

### 2.4 Reporting quality assessment

The reporting quality of the included studies was assessed independently by two reviewers using the Consolidated Health Economic Evaluation Reporting Standards 2022 (CHEERS 2022) checklist (15). The CHEERS 2022 checklist consists of 7 domains and 28 items. Each item was scored as 1 point if fully reported, 0.5 points if partially reported, and 0 points if not reported, with a maximum total score of 28 points. Disagreements between reviewers were resolved through discussion or consultation with a third reviewer.

## 3 Results

**Figure 1:**
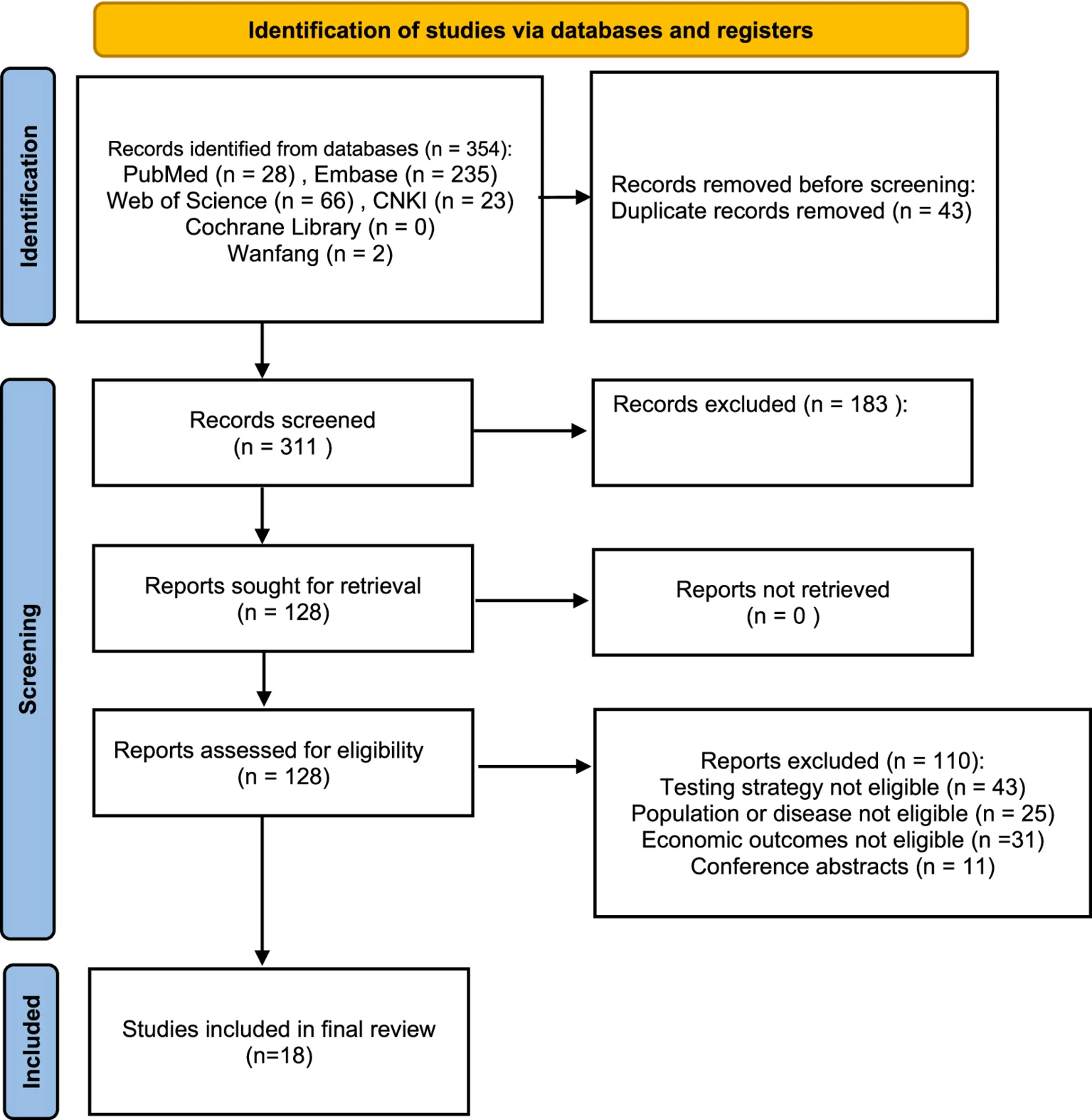
PRISMA flow diagram of study selection.

A total of 354 records were initially identified. After stepwise screening, 18 studies were ultimately included in this systematic review. The PRISMA flow diagram of study selection is shown in Figure 1.

### 3.1 Reporting quality assessment

**Figure 2:**
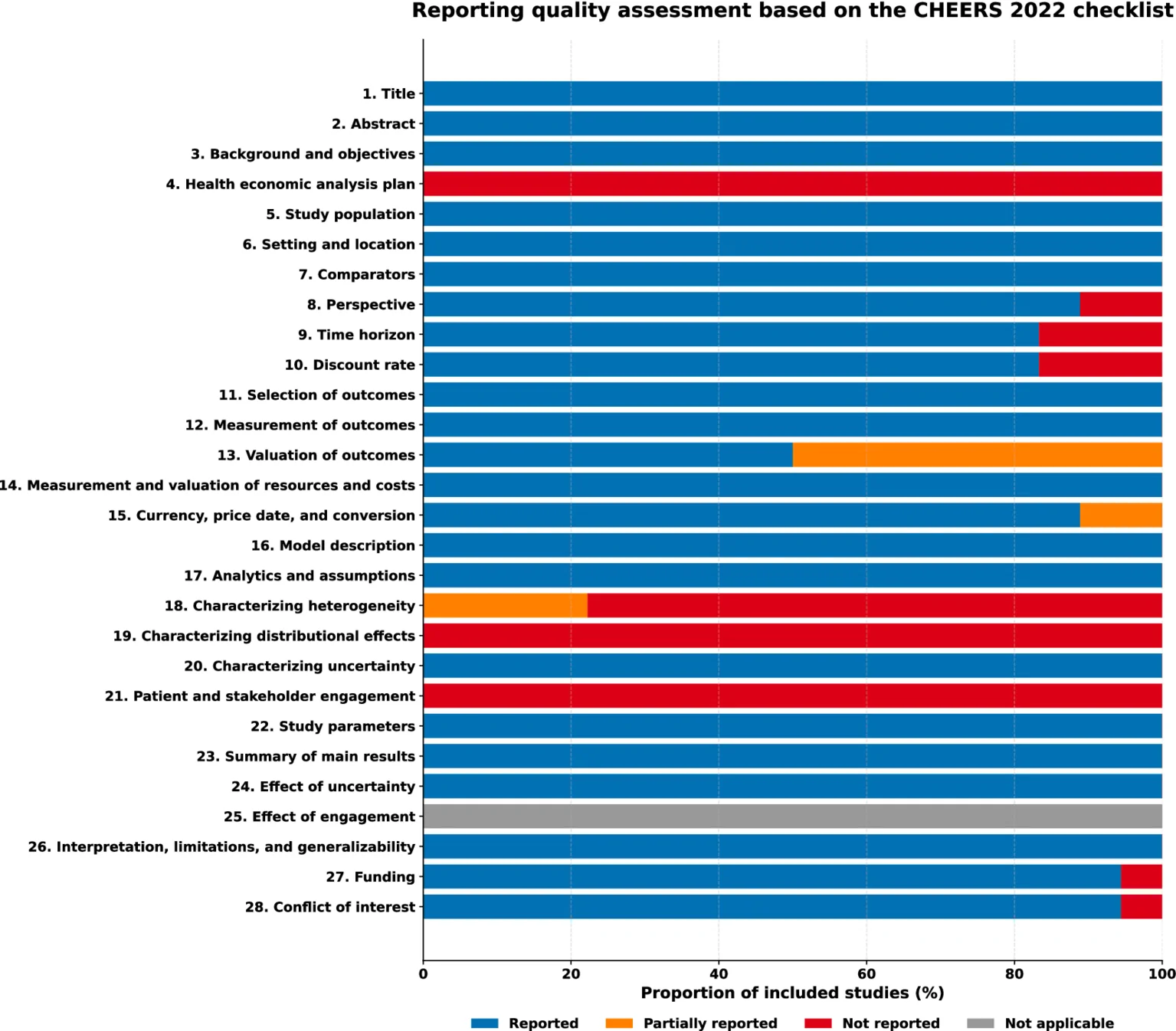
Reporting quality assessment of the included studies.

The basic characteristics of the included studies are summarized in Table 1, and the results of the reporting quality assessment are presented in Figure 2. Overall, the included studies showed relatively high reporting quality. Among the 28 CHEERS 2022 checklist items, several items were insufficiently reported, particularly study protocol, characterization of heterogeneity, distributional effects, and engagement with patients or other relevant stakeholders. None of the included studies reported a health economic analysis plan.

**Table 1:** Characteristics of the included studies.

Study	Country	Perspective	Population	Testing strategy		Cost-effectiveness conclusion
				Intervention	Comparator	
Doble et al. 2017 (16)	Australia	Health system perspective	4 L metastatic LUAD	MTS-guided targeted treatment	No further testing plus chemotherapy or BSC	Not cost-effective
Lu et al., 2018 (20)	China	Health system perspective	1 L advanced non-sq NSCLC	NGS panel- or multiplex PCR-guided crizotinib treatment	No further testing	Cost-effective with PAP; not cost-effective without PAP
Steuten et al., 2019 (28)	United States	Payer perspective	1 L advanced non-sq NSCLC	NGS-based MGPS	SMGT	Moderately cost-effective
Nadal et al., 2021 (29)	Spain	Payer perspective	1 L advanced NSCLC	Current ALK testing scenario (IHC, FISH, reflex testing, and NGS)	No further testing	Cost-effective
De Alava et al., 2022 (21)	Spain	Health system perspective	1 L advanced NSCLC	NGS panel testing	SST	Cost-effective
Wolff et al., 2022 (18)	Netherlands	NR	1 L advanced non-sq NSCLC	Parallel DNA- and RNA-based NGS plus PD-L1 IHC	Sequential single-gene testing followed by DNA/RNA NGS, plus PD-L1 IHC	Cost-effective
Zou et al., 2022 (30)	United States	Payer perspective	1 L advanced non-sq NSCLC	NGS-based testing strategies (CGP or small NGS panel) plus PD-L1	Weighted mix of SGT-based strategies plus PD-L1	Cost-effective per LY but not per QALY when post-diagnostic costs were included
Arriola et al., 2023 (33)	Spain	Healthcare institution perspective	1 L advanced NSCLC	Targeted NGS panel	SGT: parallel EGFR/ALK/ROS1 followed by sequential testing of remaining biomarkers	Cost-effective
Lemmon et al., 2023 (19)	United States	NR	1 L metastatic non-sq NSCLC	NGS	SGT (EGFR and ALK only)	More effective and associated with a lower cost per LYG than SGT
Fu et al., 2023 (22)	China	Health system perspective	NSCLC	8-gene NGS panel	qPCR (3-gene panel)	Cost-effective based on incremental diagnostic costs, but not cost-effective when total costs were included
Shiraiwa et al., 2024 (17)	Japan	Payer perspective	Metastatic NSCLC	CGP (F1CDx) after progression following first-line multiplex SGT	1. Multiplex SGT only before first-line therapy 2. Upfront CGP (F1CDx) at diagnosis	Multiple simultaneous SGT at initial diagnosis was the most cost-effective strategy; upfront F1CDx was dominated
Abbass et al., 2024 (31)	United States	Payer perspective	1 L advanced non-sq NSCLC	LP-NGS	Current empirical SGT patterns	Dominant
Kohara et al., 2024 (23)	Japan	Health system perspective	1 L advanced non-sq NSCLC	Oncomine DxTT	1. Three SGTs (EGFR/ALK/ROS1) 2. EGFR-only SGT 3.no genetic testingThree single-gene tests (EGFR/ALK/ROS1)	Oncomine DxTT was dominated; three SGTs (EGFR/ALK/ROS1) were the most cost-effective strategyDominated by three single-gene tests
Gamboa et al., 2024 (24)	Colombia	Health system perspective	1 L advanced NSCLC	CGP-based NGS	1. IHC + RT-PCR 2. FISH + RT-PCR	Cost-effective
Kang et al., 2024 (25)	South Korea	Health system perspective	1 L advanced NSCLC	NGS plus PD-1/PD-L1 screening	Guideline-based sequential SGT (ROS1, BRAF, NTRK, MET, RET, KRAS) plus PD-1/PD-L1 screening	Not cost-effective
Isla et al., 2024 (26)	Spain	Health system perspective	1 L advanced NSCLC	F1 L CDx-based liquid biopsy	non-molecular diagnosis	Cost-effective
Ou et al., 2025 (27)	Taiwan	Health system perspective	1 L advanced LUAD	Tissue NGS after negative EGFR SGT	1. Upfront tissue NGS 2. Complementary tissue and liquid NGS	Cost-effective
Turongkaravee et al., 2025 (32)	Thailand	Societal perspective	1 L advanced NSCLC	Sequential NGS testing after negative EGFR/ALK single-gene testing	Single EGFR testing	Not cost-effective

L, therapy line; CGP, comprehensive genomic profiling; FISH, fluorescence in situ hybridization; NR, not reported; BSC, best supportive care; IHC, immunohistochemistry; LP-NGS, large-panel next-generation sequencing; LUAD, lung adenocarcinoma; MGPS, multigene panel sequencing; PAP, Patient Assistance Program; MTS, multiplex targeted sequencing; non-sq, non-squamous; qPCR, quantitative polymerase chain reaction; RT-PCR, reverse-transcription polymerase chain reaction; SGT, single-gene testing; SMGT, sequential molecular genetic testing; SST, standard single-gene testing; Oncomine DxTT, Oncomine™ Dx Target Test Multi-CDx System. F1 L CDx, FoundationOne Liquid CDx.

### 3.2 Population

Most included studies enrolled patients with non-squamous NSCLC or lung adenocarcinoma, and several focused on treatment-naïve patients with advanced disease. Two studies differed from this pattern: one included patients with metastatic lung adenocarcinoma receiving fourth-line treatment (16), whereas a Japanese study included patients undergoing testing before first-line and third-line treatment, reflecting the clinical policy in Japan at that time (17).

### 3.3 Perspective

Among the 18 included studies, two did not clearly report the study perspective (18, 19). Of the remaining 16 studies, nine adopted a health system perspective (16, 20–27), five a healthcare payer perspective (17, 28–31), one a societal perspective (32), and one a healthcare institution perspective (33) (Table 1).

### 3.4 Testing and treatment strategies

Across the included studies, patients with detected actionable genomic alterations generally received matched targeted therapies, whereas those without actionable alterations received alternative treatments such as chemotherapy or immunotherapy. NGS strategies included comprehensive genomic profiling and multigene panel testing, while a small number of studies also evaluated sequential testing strategies incorporating single-gene tests. The most commonly assessed biomarkers included *ALK, EGFR, ROS1, BRAF,* and *RET*, with *EGFR* and *ALK* being the most frequently evaluated genes. Testing strategies were generally defined according to clinical guidelines, patient characteristics, and local clinical practice (Table 1).

### 3.5 Model types and structures

The 18 included studies used seven modeling approaches, comprising five single-model approaches and two hybrid approaches. The single-model approaches included decision trees, Markov state-transition models, discrete-event simulation (DES), partitioned survival models (PartSA), and microsimulation. The hybrid approaches combined a decision tree representing the diagnostic pathway with either a PartSA or Markov model to simulate long-term clinical and economic outcomes.

The decision tree–PartSA hybrid model was the most frequently used approach, accounting for 44.4% of the included studies, followed by the decision tree–Markov model at 22.2%. Studies using Markov or PartSA models generally adopted a conventional three-state structure comprising progression-free disease, progressed disease, and death. Overall, decision tree-based hybrid models were the predominant modeling approaches used to evaluate molecular testing and subsequent treatment pathways in NSCLC.

Lemmon et al. (19) developed a microsimulation model to evaluate different proportions of NGS and single-gene testing and used parametric survival distributions to estimate survival outcomes and healthcare costs under different testing strategies. Wolff et al. (18) developed a microsimulation model based on Dutch patient-level data to simulate complete diagnostic pathways involving parallel and sequential single-gene testing. The model incorporated test specificity, turnaround time, and test failure probability and linked diagnostic results to a separate treatment model to estimate clinical outcomes and costs. Abbass et al. (31) used a DES model to simulate events from diagnosis to disease progression or death, including waiting for test results and treatment initiation or switching based on test results. The model also incorporated possible NGS retesting after negative single-gene testing, allowing dynamic evaluation of the cost-effectiveness of large-panel NGS compared with current single-gene testing practice.

### 3.6 Time horizon and cycle length

Most included studies adopted a lifetime horizon to capture long-term costs and health outcomes over the disease course (21, 24, 26–30, 32, 33). Four studies used a 10-year time horizon (16, 17, 20, 22), and two adopted a 5-year horizon. Kang et al. (25) selected a 5-year horizon based mainly on the maturity of the available survival data and the survival characteristics of the target population, whereas Abbass et al. (31) used a 5-year horizon to capture the major disease course within the structure of the model.

Cycle length varied according to model type and treatment schedule. Six studies used a 1-month cycle (17, 21, 26, 29, 32, 33), two used a 3-week cycle consistent with commonly used chemotherapy or immunotherapy schedules (20, 27), and two used a 1-week cycle length (16, 25). Five studies did not use a conventional cycle length because they employed decision tree, microsimulation, or discrete-event simulation models (18, 19, 22, 28, 31). In three studies, cycle length was not clearly reported, and QALYs were estimated directly from measures such as median survival or the area under survival curves (23, 24, 30) (Table 2).

**Table 2:** Model characteristics of the included studies.

Study	Model type	Economic outcome	Time horizon	Cycle length	WTP threshold (source)	Standardized WTP threshold, 2023 Int$	Scenario analysis	Sensitivity analysis
Doble et al. 2017 (16)	Decision tree + Markov	ICER, ICUR	10 years	1 week	AUD 100,000/LY or QALY (study assumption)	Int$ 88,370/LY or QALY	No	DSA
Lu et al., 2018 (20)	Markov	ICUR	10 years	3 weeks	USD 32,000/QALY (literature-based)	Int$ 39,917/QALY	No	DSA, PSA
Steuten et al., 2019 (28)	Decision tree	ICER	Lifetime	NR	USD 150,000/LY (literature-based)	Int$ 183,624/LY	Yes	DSA
Nadal et al., 2021 (29)	Decision tree + Markov	ICER, ICUR	Lifetime	1 month	NR	NR	Yes	DSA, PSA
De Alava et al., 2022 (21)	Decision tree + PartSA	ICER, ICUR	Lifetime	1 month	EUR 22,000–30,000/LY or QALY (literature-based)	Int$ 36,282–49,476/LY or QALY	No	DSA, PSA
Wolff et al., 2022 (18)	Microsimulation model	ICUR	NR	NR	EUR 80,000/QALY (Dutch recommended threshold)	NR^a^	Yes	DSA
Zou et al., 2022 (30)	Decision tree + PartSA	ICER, ICUR	Lifetime	NR	USD 150,000/LY	Int$ 173,885/LY	Yes	DSA, PSA
Arriola et al., 2023 (33)	Decision tree + PartSA	ICER, ICUR	Lifetime	1 month	NR	NR	Yes	DSA, PSA
Lemmon et al., 2023 (19)	Microsimulation model	ICER	NR	NR	NR	NR	Yes	NR
Fu et al., 2023 (22)	Decision tree	ICER	10 years	NR	CNY 257,094/LY (3 × GDPpc)	Int$ 72,149/LY	No	DSA, PSA
Shiraiwa et al., 2024 (17)	Decision tree + Markov	ICER, ICUR	10 years	1 month	JPY 7,500,000/QALY (USD 49,500/QALY; Japanese oncology WTP threshold)	Int$ 79,235/QALY	No	DSA, PSA
Abbass et al., 2024 (31)	DES model	ICUR	5 years	NR	USD 150,000/QALY	Int$ 155,515/QALY	Yes	DSA
Kohara et al., 2024 (23)	Decision tree + PartSA	ICUR	NR	NR	JPY 7,500,000/QALY (Japanese official threshold)	Int$ 84,651/QALY	Yes	DSA
Gamboa et al., 2024 (24)	Decision tree + PartSA	ICER, ICUR	Lifetime	NR	Int$ 15,630–46,890/QALY (Colombian societal threshold)	Int$ 18,355– 55,066/QALY	No	DSA, PSA
Kang et al., 2024 (25)	Decision tree + PartSA	ICER, ICUR	5 years	1 week	KRW 50,000,000/QALY (USD 38,701/QALY; Korean oncology threshold)	Int$ 61,845/QALY	Yes	DSA
Isla et al., 2024 (26)	Decision tree + PartSA	ICER, ICUR	Lifetime	1 month	EUR 20,000–30,000/QALY (literature-based)	Int$ 31,299– 46,948/QALY	Yes	DSA, PSA
Ou et al., 2025 (27)	Decision tree + PartSA	ICUR	Lifetime	3 weeks	USD 70,000/QALY (2 × GDPpc)	Int$ 70,000/QALY	No	DSA, PSA
Turongkaravee et al., 2025 (32)	Decision tree + Markov	ICUR	Lifetime	1 month	THB 160,000/QALY (Thailand HTA guideline)	Int$ 13,021/QALY	Yes	DSA, PSA

GDPpc, gross domestic product per capita; PartSA: partitioned survival model; DSA: deterministic sensitivity analysis; PSA: probabilistic sensitivity analysis; ᵃ The WTP threshold was reported, but it was not standardized because the corresponding reference price year could not be identified.

### 3.7 WTP threshold

WTP thresholds varied substantially across the included studies and were derived from sources including published literature, national oncology-specific thresholds, gross domestic product, and gross national income. Among studies with sufficient information for standardization, QALY-based WTP thresholds ranged from Int$13,021 to Int$155,515 per QALY, whereas LY-based thresholds ranged from Int$36,282 to Int$183,624 per LY. Four studies (18, 19, 29, 33) could not be standardized because a numerical threshold or sufficient information for conversion was unavailable (Table 2).

### 3.8 Sensitivity analysis

Among the 18 included studies, 11 conducted both deterministic sensitivity analysis (DSA) and probabilistic sensitivity analysis (PSA), 17 conducted DSA, and 13 performed scenario analysis (Table 2).

### 3.9 Base-case cost-effectiveness outcomes

The results of economic evaluations of NGS-guided targeted therapy were influenced by the scope and measurement of included cost components. Across the included studies, direct medical costs generally included molecular testing and diagnostic costs, drug acquisition costs, adverse event management costs, disease management costs, and follow-up costs. A small number of studies also incorporated costs associated with avoided rebiopsy or repeat testing due to test failure (16, 18, 29, 31). One study additionally included direct non-medical costs, such as transportation costs for hospital visits (32) (Table 3).

**Table 3:** Base-case cost-effectiveness outcomes.

Study	Comparison	Currency and price year	Total cost: intervention	Total cost: comparator	Incremental cost	Effectiveness: intervention	Effectiveness: comparator	Incremental effectiveness	ICER/ICUR
Doble et al. 2017 (16)	MTS-guided targeted therapy vs. No further testing + BSC	2014 AUD	AUD193,832	AUD189,529	AUD4,303	1.466 LY	1.458 LY	0.009 LY	AUD485,199/LY
	MTS-guided targeted therapy vs. No further testing +chemotherapy	2014 AUD	AUD193,832	AUD190,019	AUD3,813	0.692 QALYs	0.684 QALYs	0.008 QALYs	AUD489,338/QALY
Lu et al., 2018 (20)	NGS-guided crizotinib vs. No further testing + pemetrexed plus cisplatin, with PAP	2016 USD	USD 31,388	USD 30,811	USD 577	0.581 progression-free LY; 1.450 overall LY; 0.780 QALYs	0.536 progression-free LY; 1.394 overall LY; 0.740 QALYs	0.045 progression-free LY; 0.056 overall LY; 0.040 QALYs	USD 14,384/QALY
	Multiplex PCR-guided crizotinib vs. No further testing + pemetrexed plus cisplatin, with PAP	2016 USD	USD 31,362	USD 30,811	USD 551	0.581 progression-free LY; 1.450 overall LY; 0.780 QALYs	0.536 progression-free LY; 1.394 overall LY; 0.740 QALYs	0.045 progression-free LY; 0.056 overall LY; 0.040 QALYs	USD 13,740/QALY
	NGS-guided crizotinib vs. No further testing + pemetrexed plus cisplatin, without PAP	2016 USD	USD 37,826	USD 30,811	USD 7,015	0.581 progression-free LY; 1.450 overall LY; 0.780 QALYs	0.536 progression-free LY; 1.394 overall LY; 0.740 QALYs	0.045 progression-free LY; 0.056 overall LY; 0.040 QALYs	USD 174,970/QALY
	Multiplex PCR-guided crizotinib vs. No further testing + pemetrexed plus cisplatin, without PAP	2016 USD	USD 37,800	USD 30,811	USD 6,989	0.581 progression-free LY; 1.450 overall LY; 0.780 QALYs	0.536 progression-free LY; 1.394 overall LY; 0.740 QALYs	0.045 progression-free LY; 0.056 overall LY; 0.040 QALYs	USD 174,327/QALY
Steuten et al., 2019 (28)	NGS-based MGPS vs. SMGT	2017 USD	USD 67,110	USD 58,297	USD 8,814	1.20 LY	1.14 LY	0.06 LY	USD 148,478/LY
Nadal et al., 2021 (29)	ALK testing scenario (IHC/FISH/reflex/NGS-guided treatment allocation) vs. No ALK testing (non-targeted therapy)	2020 EUR; cohort of 7,628 patients	EUR 858,278,111	EUR 806,959,058	EUR 51,319,053	21,233.59 LY; 14,654.68 QALYs	16,173.45 LY; 10,748.05 QALYs	5,060.14 LY; 3,906.64 QALYs	EUR 10,142/LY; EUR 13,136/QALY
De Alava et al., 2022 (21)	NGS vs. SST (short-term)	2022 EUR (cohort of 100 patients)	EUR 122,012	EUR 103,422	EUR 18,590	53 peTT; 11 peCT	23 peTT; 8 peCT	30 peTT; 3 peCT	EUR 617/peTT; EUR 6,249/peCT
	NGS vs. SST (exploratory long-term)	2022 EUR (cohort of 100 patients)	EUR 11,490,245	EUR 11,442,812	EUR 47,432	276.77 LY; 185.96 QALYs	268.87 LY; 180.74 QALYs	7.90 LY; 5.22 QALYs	EUR 6,005/LY; EUR 9,084/QALY
Wolff et al., 2022 (18)	Parallel DNA- and RNA-based NGS testing vs. sequential single-gene–based testing	EUR; price year NR; per patient	EUR 157,515	EUR 149,158	EUR 8,357	2.39 LY; 1.68 QALYs; 89.9% receiving correct treatment	2.21 LY; 1.56 QALYs; 69.4% receiving correct treatment	0.17 LY; 0.12 QALYs; 20.5% increase in correct treatment	EUR 69,614/QALY
Zou et al., 2022 (30)	NGS vs. SGT	2020 USD	USD 306,039	USD 264,100	USD 41,939	2.62 LY; 1.65 QALYs	2.34 LY; 1.48 QALYs	0.28 LY; 0.18 QALYs	USD 148,786/LY; USD 238,876/QALY
Arriola et al., 2023 (33)	NGS vs. SGT	2022 EUR (cohort of 9,734 patients)	EUR 1,276,691,348	EUR 1,255,642,769	EUR 21,048,580	28,076.11 LY; 19,317.37 QALYs	26,888.04 LY; 18,504.53 QALYs	1,188.08 LY; 812.84 QALYs	EUR 17,717/LY; EUR 25,895/QALY
Lemmon et al., 2023 (19)	NGS vs. SGT	USD; price year NR; simulated cohort of 89,000 patients	NR	NR	Cost-saving; exact total incremental cost NR	59,857 LYG	38,838 LYG	21,019 LYG	NR
Fu et al., 2023 (22)	Eight-gene NGS vs. three-gene qPCR	CNY; price year NR	CNY 134,101.36	CNY 106,661.33	CNY 27,440.03	3.27 LY; 93.81% correct detection rate	3.21 LY; 56.44% correct detection rate	0.06 LY; 37.37 percentage-point increase in correct detection	CNY 14,887.77/additional correctly detected case; CNY 97,501.18/LY based on incremental diagnostic cost; CNY 480,897.90/LY based on incremental total cost
Shiraiwa et al., 2024 (17)	Multiplex SGT only at initial diagnosis vs. SGT at initial diagnosis plus F1CDx after standard treatment	JPY; cost reference year NR	JPY 29,061,492	JPY 28,956,821	JPY 104,671	3.54 LY; 2.61 QALYs	3.46 LY; 2.56 QALYs	0.08 LY; 0.05 QALYs	JPY 1,302,987/LY; JPY 2,080,923/QALY
	F1CDx at initial diagnosis vs. multiple SGT at initial diagnosis	JPY; cost reference year NR	JPY 29,575,492	JPY 29,061,492	JPY 514,000	3.54 LY; 2.61 QALYs	3.54 LY; 2.61 QALYs	0 LY; 0 QALYs	Dominated
Abbass et al., 2024 (31)	LP-NGS vs. SGT	2022 USD	USD 539,658	USD 544,550	–USD 4,892	2.331 LY; 1.588 QALYs	2.327 LY; 1.580 QALYs	0.004 LY; 0.008 QALYs	Dominant
Kohara et al., 2024 (23)	Oncomine DxTT vs. three single-gene tests (EGFR/ALK/ROS1)	2022 JPY	JPY 12,298,099	JPY 12,083,034	JPY 215,064	21.52 QALYs	21.68 QALYs	−0.16 QALYs	Dominated
Gamboa et al., 2024 (24)	CGP vs. IHC + RT-PCR	2019 Int$	Int$380,605	Int$380,301	Int$304	2.79 LY; 1.57 QALYs	2.73 LY; 1.53 QALYs	0.06 LY; 0.04 QALYs	Int$5,017/LY; Int$7,848/QALY
	CGP vs. FISH + RT-PCR	2019 Int$	Int$380,605	Int$380,553	Int$52	2.79 LY; 1.57 QALYs	2.73 LY; 1.53 QALYs	0.06 LY; 0.04 QALYs	Int$862/LY; Int$1,348/QALY
Kang et al., 2024 (25)	NGS vs. SGT (Both PD-1/PD-L1 screening)	2022 USD	USD 87,143	USD 78,768	USD 8,375	1.895 LY; 1.190 QALYs	1.867 LY; 1.166 QALYs	0.028 LY; 0.024 QALYs	USD 300,233/LY; USD 359,405/QALY
Isla et al., 2024 (26)	Liquid CGP vs. non-molecular diagnosis	2023 EUR	EUR184,253.17	EUR181,354.86	EUR2,898.31	3.13 LY; 2.50 QALYs	2.74 LY; 2.20 QALYs	0.38 LY; 0.31 QALYs	EUR7,548.71/LY; EUR9,473.36/QALY
Ou et al., 2025 (27)	Exclusionary EGFR testing vs. upfront tissue-based NGS	2023 USD	USD 166,471	USD 165,750	USD 721	3.48 LY; 2.72 QALYs	3.43 LY; 2.67 QALYs	0.05 LY; 0.05 QALYs	USD 15,651/LY; USD 15,521/QALY
	Complementary NGS vs. exclusionary EGFR testing	2023 USD	USD 170,047	USD 166,471	USD 3,576	3.50 LY; 2.74 QALYs	3.48 LY; 2.72 QALYs	0.02 LY; 0.02 QALYs	USD 166,882/LY; USD 178,537/QALY
Turongkaravee et al., 2025 (32)	Sequential NGS testing vs. single EGFR testing	2024 THB	THB 66,283	THB 29,260	THB 37,023	30.72 life-months; 1.23 QALYs	30.36 life-months; 1.21 QALYs	0.36 life-months; 0.020 QALYs	THB 1,851,150/QALY

peTT, patients eligible for targeted therapy; peCT, patients eligible for clinical trial enrollment.

## 4 Discussion

Existing studies suggest that most model-based economic evaluations of NGS in NSCLC were conducted from a health system perspective (16, 20–27, 33) and primarily included direct medical costs (16–31, 33). These studies commonly used hybrid model structures, such as a decision tree combined with a Markov model or a decision tree combined with a partitioned survival model, to simulate the long-term economic value of using NGS to identify actionable driver alterations and guide first-line targeted therapy in patients with NSCLC. The included studies showed substantial heterogeneity in study perspective, model structure, target population, testing strategy, and comparator selection, which affected the direct comparability of cost-effectiveness outcomes across studies. Nevertheless, several consistent patterns emerged. NGS-guided strategies generally provided greater health gains than conventional testing strategies, although these additional benefits did not consistently translate into favorable cost-effectiveness across healthcare settings. The economic value of NGS appeared to depend not only on the cost of molecular testing itself, but more importantly on downstream factors such as the cost, reimbursement, and accessibility of matched targeted therapies. Other recurring drivers included the prevalence of actionable genomic alterations, testing strategy and panel scope, turnaround time, and the analytic time horizon.

Current economic evaluations of NGS have primarily compared NGS-based testing with conventional single-gene testing. However, considerable heterogeneity existed across studies in terms of testing strategies and the range of genes assessed. Some studies defined testing sequences and gene panels according to national or regional guideline recommendations (18, 25, 31), whereas others focused primarily on high-prevalence biomarkers such as EGFR and ALK (19, 20, 23, 29, 32). In addition, some studies compared NGS strategies with panels of different sizes, whereas others used no molecular testing as the comparator. These differences in intervention and comparator strategies contributed to substantial heterogeneity across studies and may partly explain the variation in reported cost-effectiveness results.

In the base-case analyses, four studies conducted in China, Japan, South Korea, and Thailand reported that NGS was not cost-effective (20, 23, 25, 32). This finding may partly reflect differences in the molecular epidemiology of NSCLC across populations and healthcare settings. East Asian patients with NSCLC have a relatively high prevalence of actionable genomic alterations, particularly *EGFR* mutations, meaning that a substantial proportion of patients may be identified using less costly single-gene testing strategies (34, 35). In such settings, the incremental value of upfront broad NGS may depend largely on its ability to identify less common actionable alterations that would otherwise remain undetected, relative to the additional costs of broader testing.

Differences in healthcare systems and reimbursement arrangements may also influence the cost-effectiveness of NGS. In several studies, economic outcomes were driven more by the cost and accessibility of matched targeted therapies than by the cost of molecular testing alone (20, 24, 25, 27, 28, 30, 32, 33). For example, Lu et al. (20) reported substantially different cost-effectiveness conclusions in scenarios with and without a patient assistance program, indicating that drug pricing and reimbursement arrangements may substantially affect the economic value of NGS-guided treatment. The analytic time horizon was another important source of variation. Many studies reporting favorable cost-effectiveness results adopted a lifetime horizon (21, 24, 26–30, 32, 33), whereas Kang et al. (25) used a 5-year horizon. A shorter time horizon may not fully capture the long-term survival benefits associated with matched targeted therapy and may therefore underestimate incremental health gains. Consistent with this interpretation, sensitivity analyses showed that ICER estimates were highly sensitive to the length of the analytic time horizon (25).

Compared with conventional single-gene testing, NGS-guided precision treatment was associated with varying degrees of gains in QALYs or LYs across the included studies (9, 36). This advantage may be partly attributable to the ability of NGS to identify a broader range of actionable genomic alterations within a single test, thereby facilitating the selection of matched targeted therapies when multiple clinically relevant alterations are considered (37, 38). However, the clinical and economic value of NGS depends largely on the availability and effectiveness of matched targeted therapies (9, 36).

Current long-term cost-effectiveness analyses may also underestimate the broader value of NGS by insufficiently capturing benefits beyond direct medical outcomes, such as fewer hospital visits, avoidance of discomfort associated with rebiopsy, and reduced patient burden related to adverse events (39). These benefits are not always fully reflected in the cost inputs or utility estimates used in existing QALY-based models (12). In addition, conventional hybrid models, such as decision tree–Markov models or decision tree–partitioned survival models, may be limited in their ability to represent the complex diagnostic and treatment trajectories of patients with cancer. More dynamic simulation approaches may better capture the sequential and time-dependent nature of molecular testing, treatment initiation, disease progression, and treatment switching (13).

Three included studies used microsimulation or discrete-event simulation models and incorporated turnaround time as a key parameter, enabling them to capture the impact of delays in obtaining molecular test results on patient outcomes (18, 19, 31). In real-world clinical practice, the median turnaround time for NGS reporting has been reported to be 8.3 weeks, which may exceed the clinically acceptable timeframe for treatment decision-making in some patients (40). When test results are substantially delayed, treatment may need to be initiated before molecular findings are available, potentially resulting in the use of chemotherapy or immunotherapy rather than matched targeted therapy in patients with actionable genomic alterations (18). In addition, approximately 7% of patients may die or experience disease progression before test results become available, thereby losing the opportunity to receive matched first-line targeted therapy (31). One-way sensitivity analyses in two studies further identified turnaround time as an important driver of ICER estimates, highlighting the importance of incorporating real-world testing delays into future economic evaluations (18, 31).

Although accumulating evidence supports the clinical and economic potential of NGS, its uptake in routine clinical practice remains suboptimal. The cost-effectiveness advantage of multigene panel sequencing over conventional testing has been most clearly demonstrated in advanced non-squamous NSCLC (5). Current NCCN guidelines recommend broad molecular profiling using large-panel NGS for eligible patients and emphasize the complementary roles of DNA- and RNA-based NGS assays in detecting clinically relevant alterations (5). Real-world evidence suggests that fewer than half of patients complete all recommended biomarker tests, although testing rates have increased over time (11, 41, 42). Even among patients who undergo molecular testing, only a limited proportion are ultimately matched to targeted therapies or clinical trials, with one study reporting a matching rate of approximately 29% (43).

In addition to technical and logistical barriers, cost remains an important obstacle to the implementation of NGS. A survey of Chinese patients with MET-altered NSCLC found that 47.1% of MET-positive patients considered the high cost of molecular testing to be an important factor influencing the use of targeted therapy (44). High testing costs and uneven reimbursement coverage may limit timely access to molecular testing and subsequent matched targeted therapy, potentially resulting in inefficient resource use and loss of clinical benefit.

Sensitivity analyses of the included economic evaluations suggested that treatment-related costs, rather than NGS testing costs alone, were among the most influential cost-related drivers of model outcomes. In the DSA results, treatment-related costs ranked among the top three influential parameters in 10 studies (17, 20, 24, 25, 27, 28, 30–33). By contrast, most models were relatively insensitive to changes in NGS testing costs, with only two studies identifying testing costs as a major sensitivity driver (21, 24). These findings suggest that the economic value of NGS-guided therapy depends not only on the cost of molecular testing but also, and potentially more importantly, on the cost, reimbursement, and accessibility of matched targeted therapies. Consistent with this interpretation, a claims-based study (41) found that patients undergoing comprehensive genomic profiling (CGP) were more likely to receive targeted therapy than those undergoing non-CGP testing or no biomarker testing, while per-patient per-month costs during first-line therapy did not differ significantly between the CGP and non-CGP groups.

The optimal panel size for first-line molecular testing in advanced NSCLC remains uncertain from an economic perspective, and further evidence is needed to clarify the necessity and economic value of rebiopsy in different clinical scenarios (45). Further economic evaluations are therefore warranted to assess the value of broad NGS strategies and inform clinical and reimbursement decision-making (11). Future studies should address several unresolved questions, including the comparative cost-effectiveness of liquid versus tissue biopsy; the economic value of different panel sizes and testing strategies, such as MGPS and CGP; the impact of molecular testing on health-related quality of life and patient preferences; and the economic value of NGS from the patient perspective.

As targeted therapies for established drivers such as EGFR and ALK continue to improve (46, 47) and effective treatment options emerge for less common actionable alterations, including MET exon 14 skipping alterations (48), a greater proportion of patients may derive meaningful survival and health-related quality-of-life benefits from molecularly matched treatment. This may, in turn, enhance the long-term clinical and economic value of NGS-guided testing. Collectively, these findings suggest that the economic value of NGS should be assessed within the entire molecular testing–treatment pathway and interpreted in the context of the local healthcare system, rather than on the basis of sequencing costs alone.

This review has several limitations. First, only studies published in Chinese or English were included, and relevant studies published in other languages may have been missed, potentially introducing language bias. Second, this review mainly included model-based economic evaluations and did not consider non-model-based studies, such as trial-based or observational economic evaluations. Third, according to the CHEERS 2022 assessment, none of the included studies reported an accessible health economic analysis plan (HEAP). Although this does not necessarily indicate the absence of a predefined analysis plan, it may reduce reporting transparency and limit the assessment of whether key analytical methods and assumptions were specified *a priori*. Fourth, heterogeneity in treatment regimens across studies may have contributed to variation in cost-effectiveness conclusions. In addition, reimbursement coverage and access to targeted therapies and molecular testing technologies vary over time and across countries or regions. Therefore, the generalizability of the findings across healthcare systems should be interpreted with caution.

## 5 Conclusion

This systematic review indicates that next-generation sequencing is a promising molecular testing strategy for non-small cell lung cancer, but its economic value is context-dependent. Despite substantial heterogeneity across studies, several consistent patterns emerged: NGS-guided strategies generally provided greater health gains, while cost-effectiveness was mainly influenced by the costs and accessibility of matched targeted therapies, the prevalence of actionable genomic alterations, testing strategy, turnaround time, and the analytic time horizon. Current evidence does not identify a universally optimal testing strategy. Future economic evaluations should therefore assess NGS within the entire molecular testing–treatment pathway and better reflect real-world factors such as tissue availability, rebiopsy, test failure, treatment-matching rates, and patient-level outcomes.
